# Intrapulpal thermal variation in standalone and dentifrice-assisted laser desensitisation at two time intervals

**DOI:** 10.1186/s12903-026-09036-5

**Published:** 2026-07-01

**Authors:** Gowri Kamath, K. Varun, Namith Rai, Rashmi Nayak, P. Kalyana Chakravarthy, Vidya Saraswathi Muliya

**Affiliations:** 1 Department of Conservative Dentistry and Endodontics Manipal College of Dental Sciences, Manipal Academy of Higher Education, Manipal, Karnataka India; 2 Renewable Energy Centre, Manipal Institute of Technology, Manipal Academy of Higher Education, Manipal, Karnataka India; 3 Department of Pedodontics and Preventive Dentistry Manipal College of Dental Sciences, Manipal Academy of Higher Education, Manipal, Karnataka India; 4 Department of Public Health Dentistry Manipal College of Dental Sciences, Manipal Academy of Higher Education, Manipal, Karnataka India

**Keywords:** Desensitisation, Dentifrice, Diode laser, Intrapulpal temperature

## Abstract

**Background:**

Dentin hypersensitivity (DH) affects a significant number of patients. Various therapeutic options have been devised to alleviate pain, including lasers, for achieving dentinal tubule occlusion. This research aimed to compare the intrapulpal temperature rise during a laser irradiation procedure for desensitisation, using laser alone and laser in combination with a dentifrice.

**Method:**

Nineteen molar tooth samples were taken, which were sectioned 2 mm below the cementoenamel junction (CEJ). Intrapulpal temperature changes were recorded using a K-type thermocouple, which was connected to a multiplexer and data logger for real-time temperature measurement at time intervals of 15 and 30 s, under stimulated pulpal microcirculation. Experiment protocols were categorised based on whether the laser was used alone (control group) or in conjunction with the dentifrice. They were further divided into two subgroups based on the duration of laser irradiation, utilising a diode laser (940 nm wavelength, 1 W power, defocused mode). The collected data were analysed to compare intrapulpal temperature changes between laser alone and laser combined with dentifrice application at both time intervals.

**Results:**

All the analysis was done using SPSS 26. There was a significant main effect of duration (*p* < 0.001), indicating a substantial change between 15 and 30 s. A significant main effect of laser irradiation was also observed (*p* < 0.001), suggesting a large difference between the laser irradiation with or without dentifrice. No significant interaction was seen between duration and laser irradiation (*p* = 0.062).

**Conclusion:**

The use of dentifrice in conjunction with laser irradiation resulted in less temperature rise compared to laser irradiation alone. However, exposure duration played a critical role in determining temperature variations.

## Background

The use of lasers in dentistry has expanded significantly, revolutionising dental practices by improving patient comfort and clinical outcomes. They are now employed in a wide range of procedures, including cavity preparation, tooth whitening, management of dentin hypersensitivity (DH), soft tissue surgeries, pulp capping, pulpotomy, laser-assisted periodontal therapy, caries detection, and aesthetic reshaping of gingival tissues [1].

DH is managed using two main categories of lasers: low-power lasers, such as helium-neon and aluminium gallium arsenide, and high-power lasers, including carbon dioxide lasers [2]. Studies have demonstrated the efficacy of diode lasers in the treatment of DH [3, 4]. However, diode lasers may increase the intra-pulpal temperature [5, 6].

Dental pulp is a specialised connective tissue that functions within a specific temperature range, helping to maintain the vitality of the tooth. The pulp reacts to an increase in temperature with an increase in blood flow [7]. Excessive heat may cause irreversible damage by interfering with its metabolic functions. It has been reported that a rise in intrapulpal temperature by 5. 6 °C (intrapulpal temperature of 42. 6 °C) may cause permanent pulp injury [8]. Numerous factors like wavelength, power and exposure time can have a potential influence on the intra-pulpal temperature [9, 10]. The temperature rise of 2. 0 °C (± 0. 5) was observed with the diode laser at a wavelength of 970 nm and a power of 2 W, whereas the temperature rise of the pulp was only 0. 1 °C(± 0. 2) when the diode laser had a wavelength of 445 nm and a power of 1 W [11].

Nano-HAp-based dentifrices have played a significant role in the reduction of DH by occlusion of microscopic dentinal tubules [12]. The use of a diode laser along with a nano-HAp dentifrice offered superior dentinal tubule occlusion compared with desensitising toothpastes alone [13]. Combination approaches using diode lasers along with desensitising agents have gained attention due to their promising clinical outcomes in the management of DH. Although studies have evaluated the thermal effects of lasers during the management of DH [14], there is a paucity of literature that assessed the intrapulpal temperature changes with increased laser exposure time and in combination with a desensitising dentifrice.

With this background and the above-identified research gaps, we aimed to assess the intrapulpal temperature using a diode laser (940 nm) assisted irradiation with or without the application of a Nano-HAp-based dentifrice at 15 and 30 s. The null hypothesis of the present study is that there is no statistically significant difference in intrapulpal temperature rise between laser irradiation used alone and laser irradiation used in combination with a dentifrice.

## Methods

The study was conducted after clearance from the Kasturba Medical College and Kasturba Hospital Institutional Ethics Committee (IEC:215/2023). This in vitro investigation utilised extracted human teeth with intact, sound dentin that were anonymised. The study aimed to evaluate and compare the rise in intrapulpal temperature during diode laser application, with and without the use of a dentifrice.

The sample size was calculated based on temperature variation outcomes reported by Guanaes et al. [15] ,which yielded an effect size of 0.95. Using a confidence level of 95% and a statistical power of 80%, the estimated sample size was 19 specimens per group. Nineteen molar teeth that were extracted for periodontal reasons were included in the study. Teeth with decay, restorations, malformations, and fractures were excluded. After cleaning and disinfection, the teeth were stored in 0. 2% Sodium Azide (*ReagentPlus*,* Sigma-Aldrich*). The root portion was sectioned 2 mm below the CEJ using a diamond disc, and the pulp chamber was irrigated with 5. 25% sodium hypochlorite for thorough cleaning.

The pulp chamber opening was enlarged to 5 mm and flattened using an inverted cone bur. A uniform buccal wall thickness of 2. 5 mm was ensured using a vernier calliper. A “through hole” was drilled from the outer surface of teeth in the palatal/lingual portion to the pulp space using a straight fissure bur for the passage of a 29-G infusion needle (*Terumo*,* USA*). An indentation was created using a straight fissure bur, which penetrated to half of its thickness on the inner surface of the buccal aspect, for the placement of K-type thermocouple wire. Type K thermocouples, composed of nickel alloys containing chromium, aluminium, manganese, and silicon, respectively, were used. The sheath surrounding the K-type thermocouple was carefully removed using a blade, and the wires, were twisted into three turns. The first contact point of the wire was positioned at the tooth indentation surface. The wire was secured in place using a bonding agent (*3 M ESPE Single Bond Universal Adhesive*,* USA)* and flowable composite resin (*Filtek Supreme Flowable Restorative*,* 3 M ESPE*,* USA*). The wires were then connected to the thermocouple extension wire, which was attached to the multiplexer. A multiplexer with a built-in thermocouple reference junction was used in conjunction with a data logger to obtain absolute temperature measurements. Keysight’s BenchLink data logger 3 software, which includes this built-in capability, was used to record the temperature every second for all samples over a duration of 15 s and 30 s. The temperatures recorded were transferred to Microsoft Excel through its built-in software interface. The real-time data were recorded at 1 Hz and expressed both graphically and as an exportable data file. From this data, the temperatures recorded at 15 and 30 s were recorded for statistical analysis.

The same set of samples was utilised for all the experiments to ensure standardisation. Two main protocols were planned. Laser irradiation procedure using the diode laser alone or in conjunction with a synthetic, water-based nano-HAp dentifrice (Acclaim toothpaste, *Group Pharmaceuticals Ltd.*,* India*). Each protocol was further divided based on the exposure time, which was either 15–30 s.

This resulted in four experimental protocols: Laser alone for 15 s and 30 s, which served as the control groups, laser with dentifrice for 15 s, and laser with dentifrice for 30 s. Intrapulpal temperature rise was measured following laser application for each.

### Stimulation of pulpal microcirculation

**Fig. 1:**
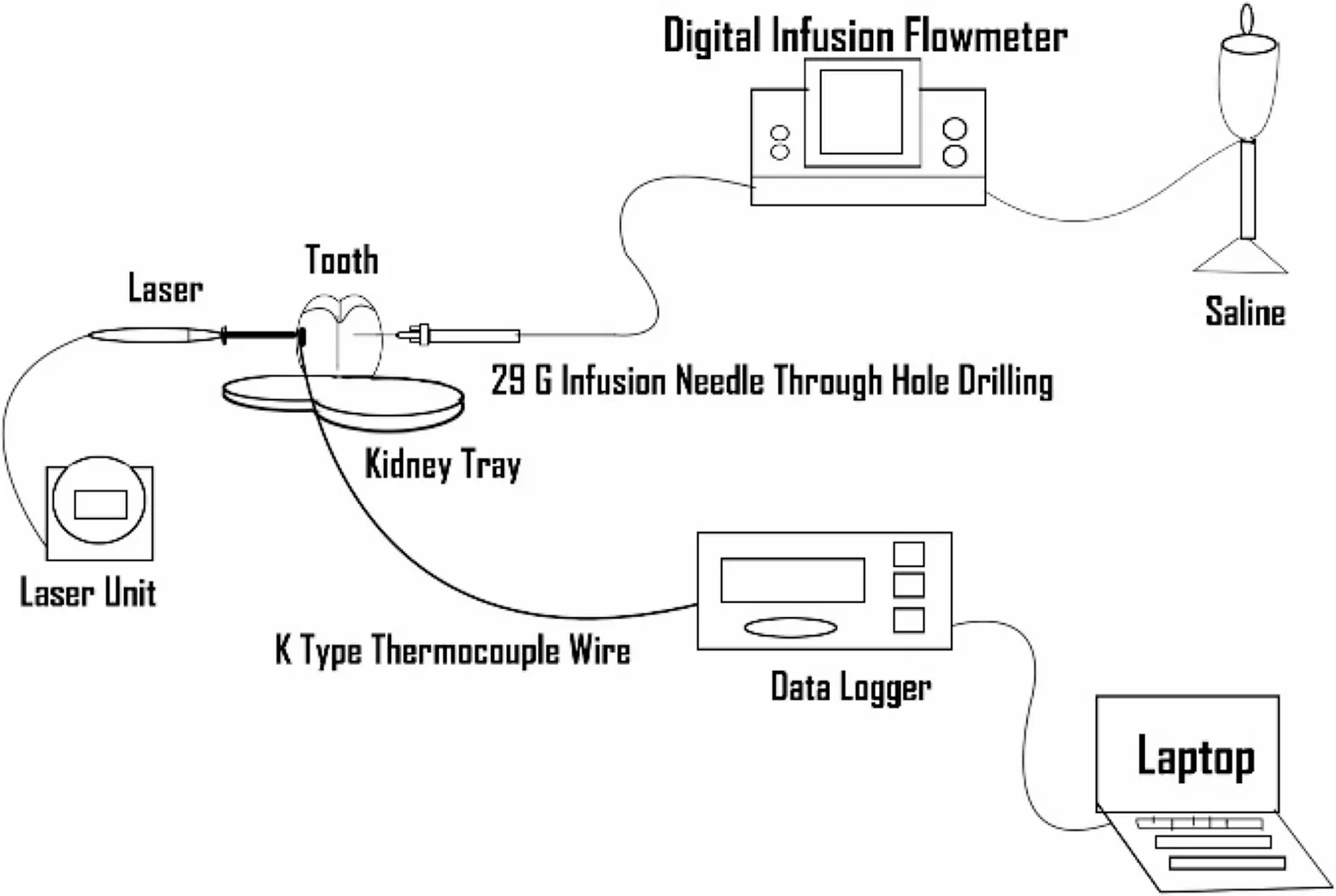
Experimental setup showing the simulated pulpal blood microcirculation apparatus

Pulpal blood microcirculation was simulated with an apparatus (Fig. 1). The apparatus allowed saline at room temperature to flow through the pulp chamber with a 29G needle to simulate pulpal vascular fluid flow [16]. A digital infusion flowmeter (*Medrena Healthcare*) was integrated into the system for controlling flow rate at a defined flow rate (0. 5 mL/min).

### Dentifrice application

A circle was drawn around a rubber stopper, which was placed on the outer surface of the buccal wall of the tooth to standardise and demarcate the area of dentifrice application. An insulin syringe (1 ml) was used to dispense 0.1 mL of dentifrice, ensuring complete coverage of the marked region. 1 mm thickness of the applied dentifrice layer was standardised and verified using a periodontal probe.

### Laser irradiation

The diode laser (*Epic X*,* Biolase Inc.*,* USA*) handpiece was held perpendicular to the enamel surface, positioned 1 mm away from the tooth surface in a non-contact mode. Subsequently, the samples were irradiated on the enamel surface with 1 W power in a continuous wave defocused mode, for 15 and 30 s, with or without the dentifrice.

All measurements were made on the same set of teeth (*n* = 19) to minimise bias during the experiment. A 1-minute interval was incorporated between measurements, of 15 and 30 s, with and without dentifrice, to allow sufficient cooling of the tissues [17], thereby minimising the risk of cumulative temperature rise. The cooling was confirmed with the reading in the data logger.

### Statistical analysis

All analyses were conducted using SPSS version 26 (SPSS Inc., Chicago, IL, USA). A P-value of < 0. 05 was considered statistically significant. Normality was tested using the Shapiro-Wilks test. A two-way repeated-measures ANOVA was performed to evaluate the role of duration of exposure and laser irradiation with or without dentifrice on intrapulpal temperature rise.

## Results

The mean intrapulpal temperature rise during laser irradiation with or without dentifrice is depicted in Table 1.

**Table 1:** Mean intrapulpal temperature rise during laser irradiation with or without dentifrice

	Laser		Laser with toothpaste	
	Mean	SD	Mean	SD
15 seconds	2.66	0.85	1.31	0.62
30 seconds	3.43	1.12	1.62	0.63

**Fig. 2:**
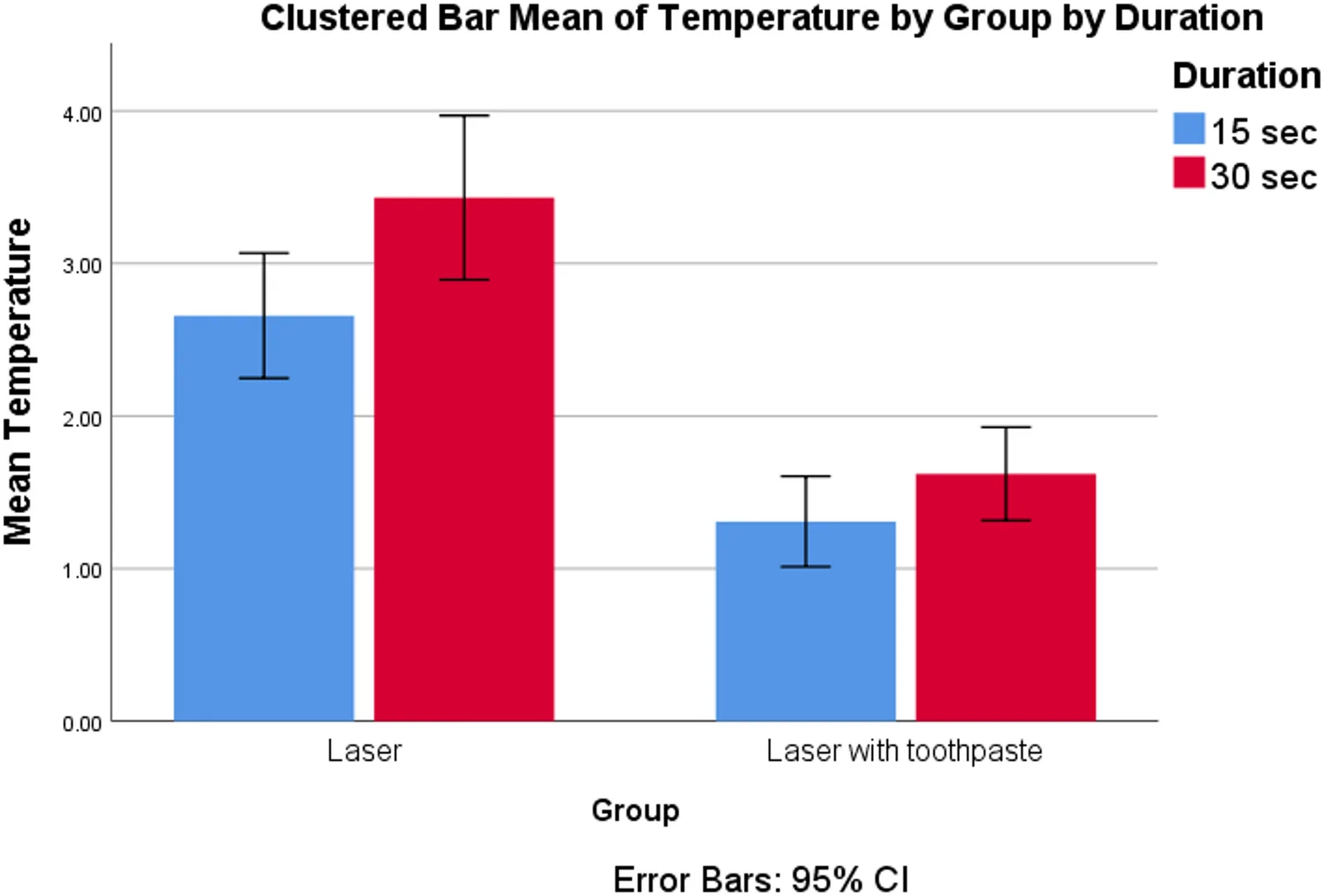
Comparison of the mean temperature rise in both experimental protocols with and without dentifrice at 15 and 30 seconds

A two-way repeated measures ANOVA was conducted to evaluate the effects of duration of exposure and laser irradiation with or without dentifrice on the intrapulpal temperature rise. There was a significant main effect of duration (*p* < 0.001, effect size = 0.364), indicating a substantial change between 15 and 30 s. A significant main effect of laser irradiation was also observed (*p* < 0.001, effect size = 0.544), suggesting a large difference between the laser irradiation with or without dentifrice. The interaction between duration and laser irradiation was not statistically significant (*p* = 0.062, effect size = 0.093), suggesting that the temperature changed over time in a similar way for both laser irradiation with or without dentifrice (Fig. 2).

## Discussion

The present study evaluated intrapulpal temperature rise during laser irradiation procedures using a laser alone and a laser in combination with a desensitising dentifrice. The aim was to determine the dentifrice’s potential in mitigating thermal buildup and to assess the impact of exposure time on intra-pulpal temperature. The present findings reinforce existing evidence regarding the thermal safety of laser therapy in the management of DH. There was a significant increase in intrapulpal temperature with the application of the laser alone, compared to the laser with dentifrice. Additionally, increasing laser exposure time from 15 to 30 s resulted in higher intrapulpal temperature values in both experimental protocols.

The application of lasers in the management of DH has garnered interest owing to their efficacy in effectively sealing dentinal tubules. Lasers can mitigate DH through two primary mechanisms: (i) an immediate effect characterised by the reduction of pain symptoms, and (ii) a delayed effect involving enhanced cellular metabolic activity, stimulation of odontoblast-like cell production, formation of reparative dentin, and the natural occlusion of dentinal tubules [18]. The rapid desensitising therapeutic effect of lasers may be attributed to mechanisms such as the diode laser’s ability to suppress nerve conduction within the pulp and induce alterations on the surface of exposed dentin [19]. Laser irradiation disrupts the crystalline arrangement and causes the dentin tissue to melt. A 940-nm diode laser has shown a significant decrease in DH pain immediately and fourteen days post-treatment [20]. Hence, a 940-nm diode laser was selected for this study.

Based on previous evidence demonstrating enhanced dentinal tubule occlusion with the combined use of diode laser and dentifrice [13], a dentifrice containing nano-HAp was selected for the present study due to its potential to facilitate apatite formation not only on the dentinal surface but also within the tubules to depths of approximately 10–15 μm, contributing to enhanced and prolonged occlusion [21].

A major concern regarding the use of lasers is the risk of inducing a rise in intrapulpal temperature [11, 19], which can lead to irreversible pulpal damage. A temperature elevation by 5. 6 °C has been reported to result in irreversible pulpitis [8, 15]. This threshold has subsequently been adopted as the maximum temperature rise that the dental pulp can tolerate without sustaining irreversible damage. Several studies have shown that if the temperature rises by 10 °C above body temperature for one minute, the alveolar bone and periodontal tissues will undergo irreversible damage [22].

Pulpal blood flow plays a key role in dissipating heat and reducing intrapulpal temperature. In previous research, pulpal flow was simulated at rates of 1 or 0. 5 mL/min. Therefore, normal saline with a flow rate of 0. 5 mL/min was used in the present study to mimic physiological pulpal microcirculation [16]. To mitigate the methodological gap of the uncontrolled flow rate of the coolant in earlier studies [6, 9], a digital flowmeter was used in this study, which maintained a uniform flow rate throughout the experiment period. Additionally, care was taken to prevent direct contact between the saline and the exposed thermocouple tip by sealing it with composite resin [6].

Inherently, due to their low thermal conductivity and diffusivity, enamel and dentin serve as natural insulating barriers, offering protection to the pulp against harmful thermal stimuli [7]. In the present study, variability in dentinal thickness among different teeth was controlled by utilising the same set of teeth across all experimental groups, thereby eliminating potential bias.

A diode laser power setting of 1 W was selected based on its well-documented ability to achieve optimal dentinal tubule occlusion while preserving pulpal integrity [23]. According to Umana et al., both 0. 8 W and 1 W in continuous mode were sufficient to effectively obliterate dentinal tubules [24], without causing surface damage, as confirmed by SEM analysis [25]. In contrast, higher settings, such as 2 W, resulted in areas of dentin destruction aligned with the results of Gutknecht et al. [26].

While previous studies have demonstrated the efficacy of 15-second diode laser exposures for dentin desensitisation [27], extending the exposure time to 30 s was considered to potentially enhance treatment outcomes. It was observed that longer exposure durations resulted in greater desensitising effects, suggesting a dose-response relationship between exposure time and treatment efficacy [28].

Various temperature-measuring tools have been utilised in comparable studies, such as thermocouples, infrared cameras, calorimeters, and differential thermal analysis devices [19]. Due to their reliability and sensitivity in detecting internal temperature variations [7, 19], thermocouples are considered the most effective method for assessing intrapulpal temperature rises, as alternative techniques present certain limitations.

The present study demonstrated a significant association between laser exposure time and pulpal thermal response. In the laser-only group, extending the laser irradiation time from 15 s to 30 s resulted in a significant increase in intrapulpal temperature. Similarly, a difference in temperature rise was observed when exposure time was doubled, even in the presence of a dentifrice. These findings confirm that the duration of laser irradiation plays a pivotal role in thermal changes.

Carrasco et al. utilised a 970 nm diode laser at 40 mW for 20 s and showed a minimal temperature rise of just 0. 2 °C when bleaching gel was applied, suggesting the potential of adjunctive materials in modulating thermal effects during laser used [29]. In the present study, comparisons across equivalent durations in both cases confirm that the dentifrice substantially reduces the rise in intrapulpal temperature. The dentifrice acts as a thermal moderator, thereby contributing to a reduction in temperature rise during laser application.

The present study is limited by its in-vitro design and the use of a single laser wavelength.

## Conclusion

It can be concluded that the combination of a diode laser(940 nm) with a dentifrice at shorter exposure times appears to offer a safer thermal profile, highlighting the importance of adjunctive materials and controlled exposure durations in preventing potential thermal injury to the pulp during clinical desensitisation treatments using diode lasers.

## Data Availability

The corresponding author can provide the datasets used and/or analyzed on request.

## Abbreviations

DH: Dentinal Hypersensitivity
Nano-HAp: Nano-hydroxyapatite
CEJ: Cementoenamel junction
